# LINC00520 is induced by Src, STAT3, and PI3K and plays a functional role in breast cancer

**DOI:** 10.18632/oncotarget.11962

**Published:** 2016-09-10

**Authors:** Whitney S. Henry, David G. Hendrickson, Francisco Beca, Benjamin Glass, Marianne Lindahl-Allen, Lizhi He, Zhe Ji, Kevin Struhl, Andrew H. Beck, John L. Rinn, Alex Toker

**Affiliations:** ^1^ Department of Pathology, Beth Israel Deaconess Medical Center, Harvard Medical School, Boston, MA, USA; ^2^ Department of Stem Cell and Regenerative Biology, Harvard University, Cambridge, MA, USA; ^3^ Broad Institute of MIT and Harvard, Cambridge, MA, USA; ^4^ Department of Biological Chemistry and Molecular Pharmacology, Harvard Medical School, Boston, MA, USA

**Keywords:** lncRNA, Src, PI3K, breast cancer, LINC00520

## Abstract

Long non-coding RNAs (lncRNAs) have been implicated in normal cellular homeostasis as well as pathophysiological conditions, including cancer. Here we performed global gene expression profiling of mammary epithelial cells transformed by oncogenic *v-Src*, and identified a large subset of uncharacterized lncRNAs potentially involved in breast cancer development. Specifically, our analysis revealed a novel lncRNA, LINC00520 that is upregulated upon ectopic expression of oncogenic *v-Src*, in a manner that is dependent on the transcription factor STAT3. Similarly, LINC00520 is also increased in mammary epithelial cells transformed by oncogenic PI3K and its expression is decreased upon knockdown of mutant *PIK3CA.* Additional expression profiling highlight that LINC00520 is elevated in a subset of human breast carcinomas, with preferential enrichment in the basal-like molecular subtype. ShRNA-mediated depletion of LINC00520 results in decreased cell migration and loss of invasive structures in 3D. RNA sequencing analysis uncovers several genes that are differentially expressed upon ectopic expression of LINC00520, a significant subset of which are also induced in *v-Src*-transformed MCF10A cells. Together, these findings characterize LINC00520 as a lncRNA that is regulated by oncogenic Src, *PIK3CA* and STAT3, and which may contribute to the molecular etiology of breast cancer.

## INTRODUCTION

Cancer is largely driven by genetic alterations, which lead to the deregulation of gene networks that typically govern normal cellular homeostasis. Recent studies have implicated long non-coding RNAs (lncRNAs) in a diverse array of human cancers [1–4]. Surprisingly, a large number of these non-coding transcripts are found in genomic regions that experience frequent mutation or somatic copy number alterations [5]. In addition, many lncRNAs are transcriptionally regulated by major oncogenes and tumor suppressors including *c-Myc* and *p53* respectively [6, 7].

Gene expression profiling of various disease model systems has proven to be a powerful approach for identifying candidate lncRNAs implicated in cancer. The first cancer-associated lncRNAs to be identified using differential expression profiling of prostate tumors and normal tissue, were prostate cancer associated 3 (*PCA3*, also called *DD3*) which is currently used as a biomarker for prostate cancer [8, 9], and prostate-specific transcript 1 (*PPCGEM1*) which is implicated in androgen receptor transcriptional activation [10, 11]. Differential expression profiling has also led to the discovery of the nuclear lncRNA metastasis-associated lung adenocarcinoma transcript 1 (*MALAT1*), as one of the first lncRNAs to be ascribed a role as a potential prognostic biomarker for lung cancer survival [12].

Collectively, lncRNAs may act in either a tumor suppressive or oncogenic capacity to modulate cellular phenotypes associated with malignancy [13]. One of the best-characterized cancer-associated lncRNAs is *HOTAIR*. This lncRNA acts as a molecular scaffold for Polycomb Repressive Complex 2 (PRC2) and Lysine-Specific Demethylase 1 (LSD1) to facilitate epigenetic silencing of specific gene loci and promotes breast cancer metastasis [1]. Furthermore, expression of *HOTAIR* is also associated with poor survival [1]. *ANRIL* is another lncRNA implicated in cancer. Expression of this antisense non-coding RNA in prostate cancer cells, results in the transcriptional repression of the *INK4n/ARF/INK4a* tumor suppressor genes, which regulate cell cycle progression and senescence [14]. Similarly, in melanoma cells, RNAi-mediated knockdown of the highly expressed lncRNA SPRY4-IT1 results in defects in cell growth and induction of apoptosis [15].

In spite of these examples, less than 1% of the identified human lncRNAs have been characterized [16]. Our understanding of lncRNA biology is far from complete and the identification, regulation and functional characterization of lncRNAs involved in breast cancer pathogenesis may provide novel opportunities for differential diagnoses and therapeutic interventions. Here we identify the novel lncRNA LINC00520 in breast cancer using two independent systems of cellular transformation driven by oncogenic *v-Src* and mutant *PIK3CA*, respectively. We further demonstrate that LINC00520 expression is clinically relevant and is preferentially associated with basal-like breast cancer. We also investigate the transcriptional regulation of LINC00520 and provide evidence for its role in breast cancer development.

## RESULTS

### Identification and transcriptional regulation of LINC00520 in a model of Src-induced transformation of mammary epithelial cells

In order to assemble a comprehensive list of lncRNAs that are potentially implicated in breast cancer, we systematically surveyed the transcriptome of a well-characterized immortalized mammary epithelial MCF10A cell line model containing a tamoxifen-inducible Src oncoprotein (*v-Src*). Previous studies using this model system have demonstrated that ectopic expression of *Src* results in multiple features associated with cellular transformation, including colony formation in soft agar, increased migration and invasion and tumor formation capability in immunocompromised mice [17]. Furthermore, Src-induced transformation has been demonstrated to drive an onset of molecular events that involve epigenetic alterations leading to changes in gene expression networks [17].

**Figure 1:**
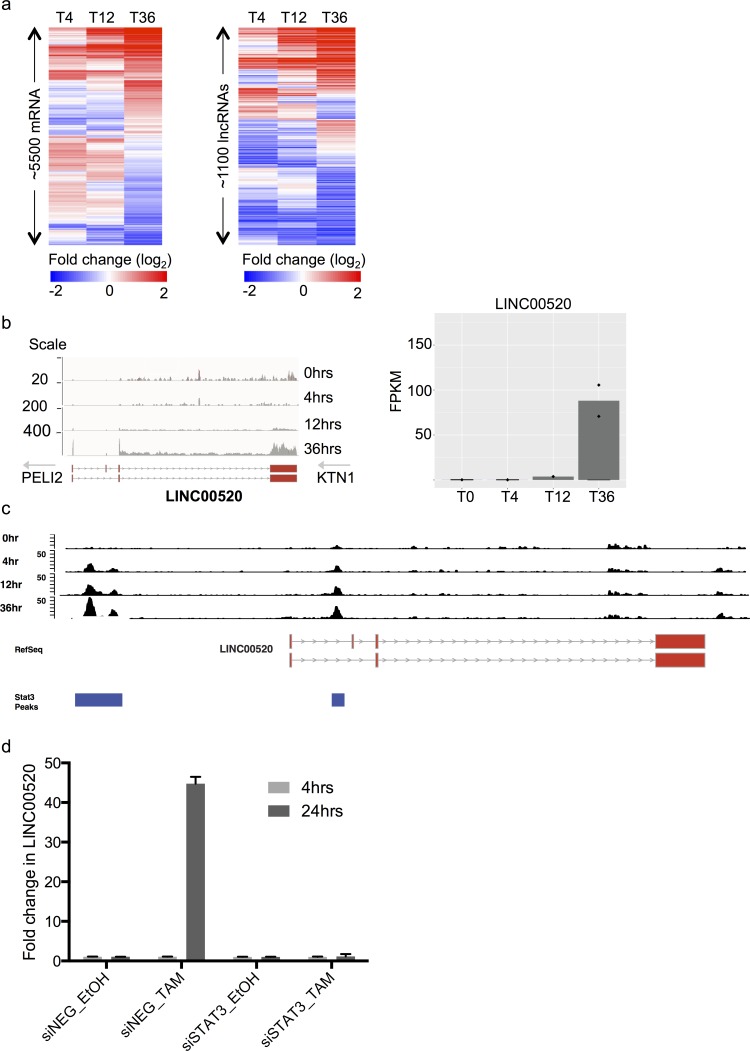
Identification and transcriptional regulation of LINC00520 in a model of Src-induced transformation of mammary epithelial cells **a.** Heat maps showing subset of protein coding genes and long non-coding RNAs that are differentially expressed at 4,12 and 36 hours post Src induction, in MCF10A cells. **b.** RNA Sequencing, relative expression of LINC00520 at various time-points post Src induction in immortalized mammary epithelial MCF10A cells. FPKM, fragments per kilobase of transcript per million mapped reads. **c.** STAT3 ChIP enrichment in MCF10A cells post Src induction, at the LINC00520 locus. **d.** Expression of LINC00520 following siRNA-mediated depletion of STAT3 in MCF10A-Src transformed cells. Transcript levels were determined by qRT-PCR and normalized to GAPDH. Values represent the average of three technical triplicates.

To explore the transcriptome of MCF10A cells upon Src induction, we collected RNA before (T0) and after Src induction at 4, 12, and 36 hours (T4,T12,T36) and performed RNA-sequencing. Differential expression analysis revealed thousands of protein coding genes and hundreds of differentially regulated non-coding transcripts (Figure 1A). As expected, we observed concordant overlap with the transcriptional signature previously defined in this system [17]. To identify lncRNAs with oncogenic potential we focused on a subset of the ncRNAs whose transcript levels are robustly increased upon *v-Src* induction (Figure 1A).

To pare down the number of potential candidates, we ordered the transformation-induced lncRNAs by fold induction as well as final transcript abundance at 36 hours. We reasoned that a potent oncogenic lncRNA would display both strong induction AND high expression. Topping both criteria was LINC00520, an uncharacterized lncRNA that displayed both striking induction (> 30 fold) and abundance of ~ 80 FPKM at 36 hours (Figure 1B). Consequently, LINC00520 ranked in the ~95 percentile of expressed genes which is at the high end of both reported lncRNA and coding expression regimes. Subsequent analyses on LINC00520 indicates that it resides ~112kb from the kinesin receptor *KTN1* and ~ 321kb from the Pellino E3 ubiquitin ligase family member 2, *PELI2* (Figure 1B). In support of LINC00520 being an independent transcript, we note that LINC00520 is transcribed in the opposite direction to either flanking gene. In addition, transcript structural analysis reveals that LINC00520 undergoes splicing and contains 3-4 exons depending on the isoform type (Figure 1B).

### LINC00520 is regulated by STAT3 in Src-transformed cells

Since the transcription factor signal transducer and activator of transcription 3 (STAT3) plays a critical role in Src-induced transcriptional responses during cellular transformation [17], we analyzed published chromatin immunoprecipitation (ChIP) data performed in the MCF10A Src-induced cells to determine whether STAT3 directly binds to the LINC00520 promoter [18]. An enrichment of STAT3 binding to the LINC00520 promoter region is observed as early as 4 hours post Src induction, with a significant increase at 36 hours. This coincides with an increase in LINC00520 transcript levels at this time point (Figure 1C). Moreover, depletion of STAT3 with siRNA abolishes Src-induced upregulation of LINC00520 (Figure 1D). Taken together, these data implicate STAT3 in the transcriptional regulation of LINC00520 during cellular transformation of mammary epithelial cells driven by oncogenic Src.

### LINC00520 is regulated by the PI3K pathway

**Figure 2:**
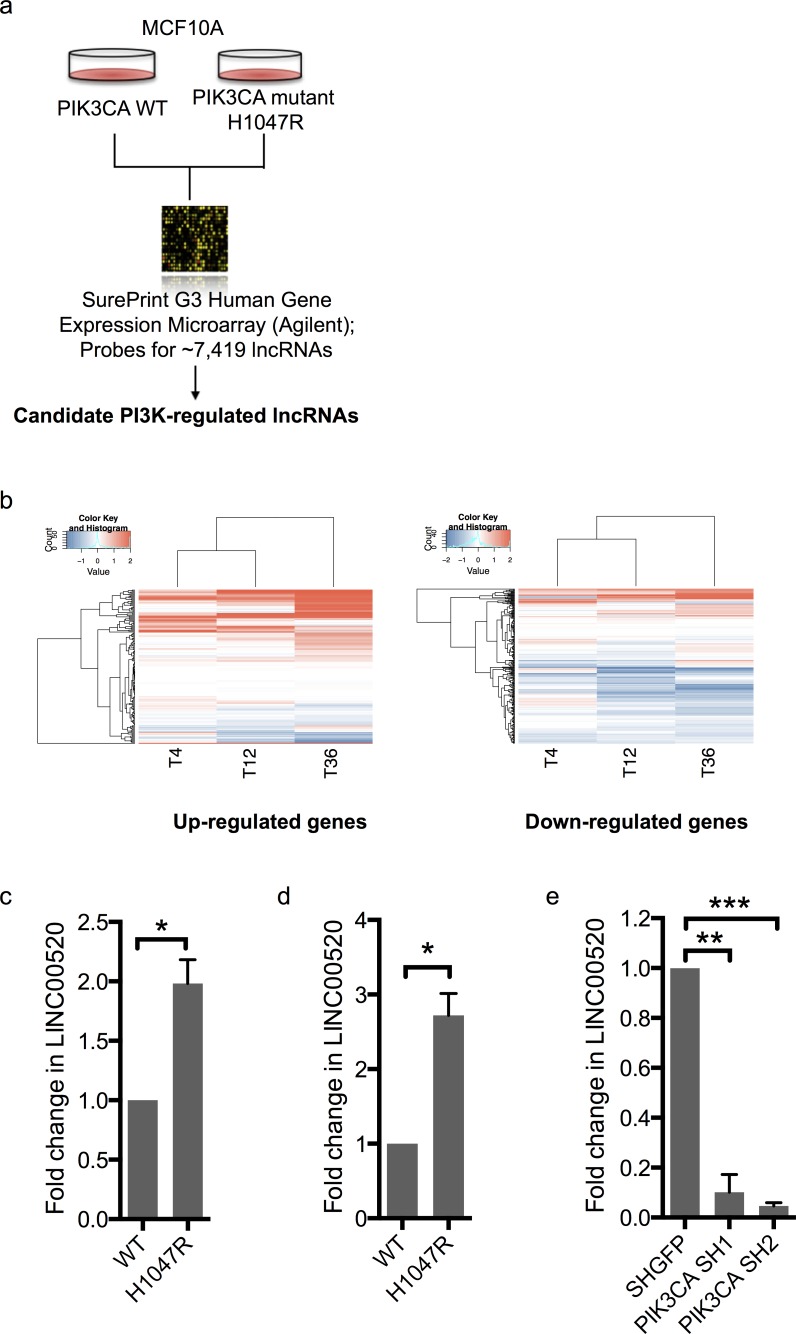
LINC00520 is regulated by the PI3K pathway **a.** Schematic of MCF10A system used to identify PI3K regulated genes involved in transformation. **b.** Heat map of gene expression values from Src-transformed MCF10A cells for genes that increased (left) or decreased (right) by two fold in the mutant *PIK3CA*-transformed MCF10A cells. Quantitative RT-PCR, showing relative expression of LINC00520 in: **c.** MCF10A cells expressing wild-type *PIK3CA* (WT) compared to mutant *PIK3CA H1047R*, **d.** Isogenic MDA-MB-231 cells expressing wild-type *PIK3CA* (WT), and mutant *PIK3CA H1047R* respectively and **e.** shRNA-mediated knockdown of *PIK3CA* in SUM159-PT cells. Transcript levels were normalized to GAPDH. Data represents average of three independent experiments ± the SEM. Statistical significance was determined using paired Student's *t*-test, * *p* < 0.05, ** *p* < 0.01, ***p < 0.001.

To investigate if LINC00520 plays a broader role in transformation we turned to an orthogonal model using MCF10A mammary epithelial cells expressing oncogenic mutants of *PIK3CA*. The PI3K pathway is frequently hyperactivated in breast cancer mainly due to recurrent somatic mutations in *PIK3CA*, the gene that encodes the p110α catalytic subunit of PI3K, or *via* loss of the tumor suppressor Phosphatase and Tensin Homolog (*PTEN)* [19]. As previously reported, oncogenic *PIK3CA* (*H1047R)* induces cellular transformation as indicated by the increase in colony formation in soft agar compared to wild-type *PIK3CA* (Supplementary Figure 1) [20]. We collected gene expression data from *PIK3CA* (*H1047R)* MCF10A cells and compared this to WT p110α using microarrays (Figure 2A). We found that many differentially expressed genes in the *PIK3CA* (*H1047R)*-expressing cells are also differentially expressed in Src-transformed MCF10A cells, including LINC00520 (Figure 2B). We verified LINC00520 expression using qRT-PCR and again found that increased LINC00520 transcript levels are observed in MCF10A cells expressing mutant *PIK3CA* (*H1047R)* relative to wild-type *PIK3CA-*expressing cells (Figure 2C). Likewise, a similar upregulation of LINC00520 is observed in isogenic MDA-MB-231 cells expressing *PIK3CA* (*H1047R)* relative to wild-type (Figure 2D). By contrast, LINC00520 is downregulated upon depletion of *PIK3CA* in SUM159-PT (mutant *PIK3CA H1047L*) breast cancer cells (Figure 2E). Together these data suggest that LINC00520 is regulated downstream of oncogenic PI3K.

### LINC00520 is upregulated in human tumors and enriched in basal-like human breast carcinomas

**Figure 3:**
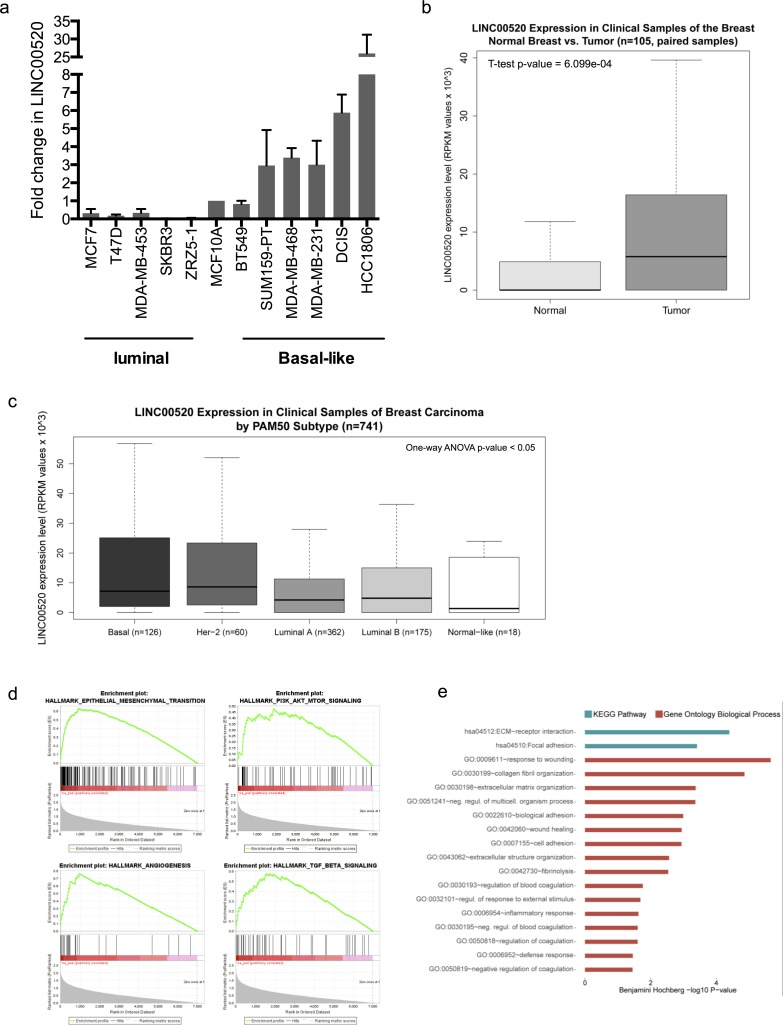
Expression Analysis in Clinical Samples of Human Breast Invasive Carcinoma **a.** Relative expression of LINC00520 in a panel of breast cancer cell lines, compared to MCF10A cells. Expression was determined by qRT-PCR and normalized to GAPDH. Values are representative from three independent experiments. **b.** Breast carcinoma clinical samples show higher expression of LINC00520 compared to matched normal breast samples from TCGA dataset (*n* = 105). **c.** In breast carcinoma clinical samples (*n* = 741) LINC00520 expression level differs according to the intrinsic molecular subtype. RPKM, reads per kilobase of transcript per million mapped reads. **d.** Gene Set Enrichment analysis (GSEA) of basal carcinomas with high expression of LINC00520. Four of the most associated gene sets, of the 50 Hallmark gene sets of the Broad Institute's MSigDB collection, 11 gene set S were significantly upregulated at an FDR < 1% and 26 at an FDR < 5% using Hallmark gene set collection. **e.** Top-ranked Functional Gene Ontology Biological Process and KEGG Pathway terms using DAVID. All represented terms were associated with the transcriptomic signature of basal breast carcinomas with high LINC00520 expression (Benjamini Hochberg *p*-value of < 0.05).

It has been reported that lncRNAs tend to display tissue specificity. Therefore, we next examined the expression of LINC00520 in a panel of breast cancer cell lines with defined genetic alterations and molecular subtypes. Interestingly, we find a preferential upregulation of LINC00520 in basal-like, triple-negative breast cancer cell lines (Figure 3A), many of which display a high metastatic potential and poor prognosis.

To better understand this finding, we analyzed LINC00520 expression in breast carcinoma clinical samples. Using RNA-sequencing data, we found that LINC00520 is upregulated in human breast tumors (Figure 3B) and it is particularly enriched in basal-like and *HER2* intrinsic molecular subtypes (Figure 3C). Furthermore, within the basal-like subtype, we found 166 differentially expressed genes (of 13, 015 transcripts) in samples with high LINC00520 expression (Supplementary Figure 2). Gene set enrichment analysis (GSEA) (based on a pre-ranked gene list using SAM's d-statistic) revealed that the expression of LINC00520 in basal-like breast cancer was significantly associated with upregulation of 26 of the Broad Institute's Molecular Signatures Database (mSigDB) Hallmark gene sets at a FDR < 5% (Supplementary Figure 3), including gene sets related to activation of the PI3K/AKT/mTOR pathway and epithelial-mesenchymal-transition (EMT) (Figure 3D). Consistent with this finding, functional analysis, indicates that many of the differentially expressed genes in basal-like carcinomas of the breast with high LINC00520 expression were primarily implicated in processes involved in cell adhesion, extracellular matrix remodeling, and wound healing (Figure 3E) and may therefore provide insight into potential roles of LINC00520 in breast cancer.

### Depletion of LINC00520 blocks breast cancer cell migration

**Figure 4:**
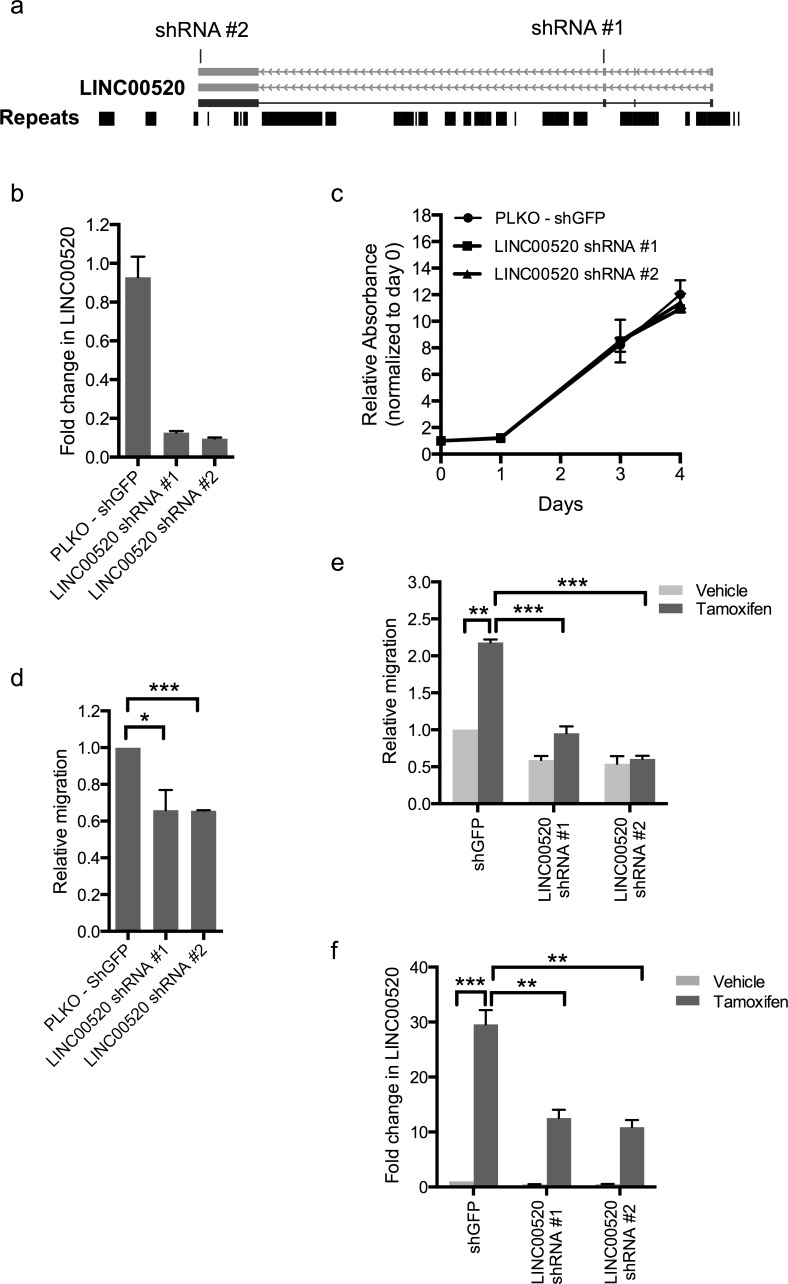
Depletion of LINC00520 blocks breast cancer cell migration **a.** Schematic showing regions targeted by LINC00520 shRNA#1 and shRNA#2. **b.** Validation of downregulation of LINC00520 in SUM159-PT cells used for proliferation and migration assays. **c.** Proliferation assay using SUM159-PT cells expressing two different LINC00520 shRNA constructs. Data represents average of two biological replicates. **d.** Transwell migration assay of SUM159-PT cells expressing two different LINC00520 shRNA constructs. **e.** Transwell migration assay of MCF10A-Src inducible cells expressing two different LINC00520 shRNA constructs. Cells were induced with vehicle (ethanol) or Tamoxifen for 48hours prior to migration. **f.** Validation of downregulation of LINC00520 in MCF10A-Src inducible cells used for migration assays. Data represents average of three biological replicates ± the SEM. Statistical significance was determined using unpaired Student's t test, * *p* < 0.05, *** *p* < 0.001.

**Figure 5:**
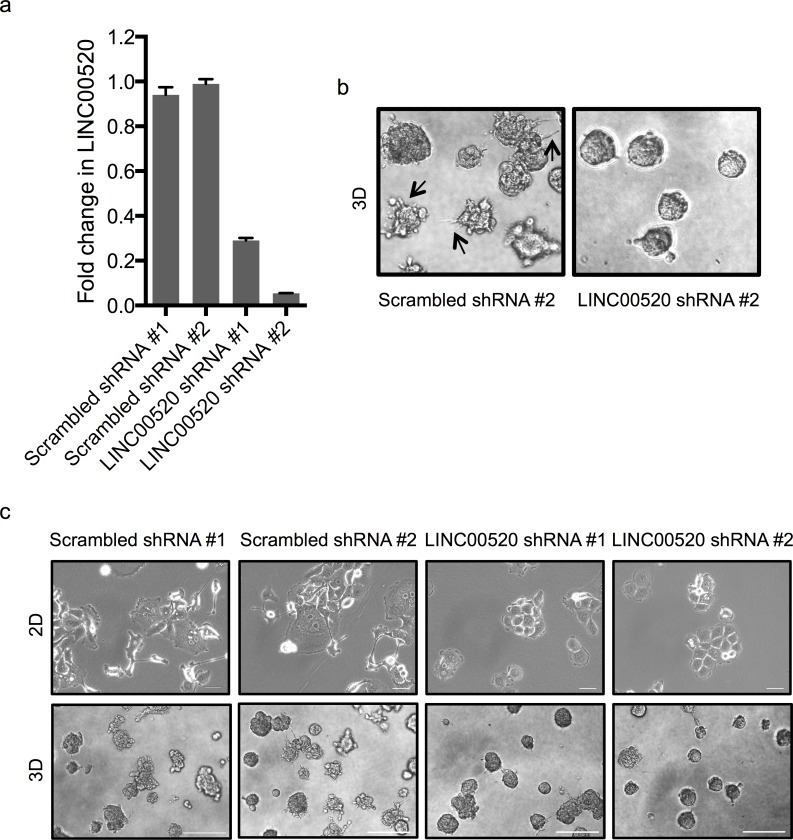
Depletion of LINC00520 affects the integrity of invasive protrusions **a.** Quantitative RT-PCR validation of silencing of LINC00520 in MCF10DCIS cells. Transcript levels were normalized to GAPDH and fold change was calculated relative to scrambled shRNA #1. **b.** Representative phase-contrast images of MCF10DCIS acini infected with lentiviral scrambled or LINC00520 shRNA grown in 3D Matrigel. Images displayed were taken on day 5. **c.** Morphological effects of silencing of LINC00520 in MCF10DCIS cells grown in (Top) 2D, scale bar = 100μm and (Bottom) in 3D Matrigel cultures. For 3D, images shown were taken at day 5, scale bar = 500μm.

In order to assess the biological role of LINC00520 in breast cancer, we performed shRNA-mediated loss-of-function studies in basal-like breast cancer cell lines containing endogenous levels of LINC00520. Two distinct shRNA constructs, each targeting a distinct exon of LINC00520, were cloned (Figure 4A). Approximately 70-90% silencing efficiency was observed by qRT-PCR in all cell lines tested including SUM159-PT cells (Figure 4B), MCF10A-Src cells (Figure 4F) and MCF10DCIS cells (Figure 5A). We then investigated the effects of LINC00520 depletion on cell proliferation. We observed no significant effects on the proliferation of SUM159-PT cells upon depletion of LINC00520 (Figure 4C). By contrast, depletion of LINC00520 leads to a decrease in transwell migration of SUM159-PT cells (Figure 4D) and MCF10A-Src transformed cells (Figure 4E). Similarly, a reduction of invasive protrusions from MCF10DCIS spheroids grown in Matrigel is observed upon depletion of LINC00520 (Figure 5B-5C). These data implicate LINC00520 in cell migration and invasion.

### Overexpression of LINC00520 results in alterations in global gene expression

**Figure 6:**
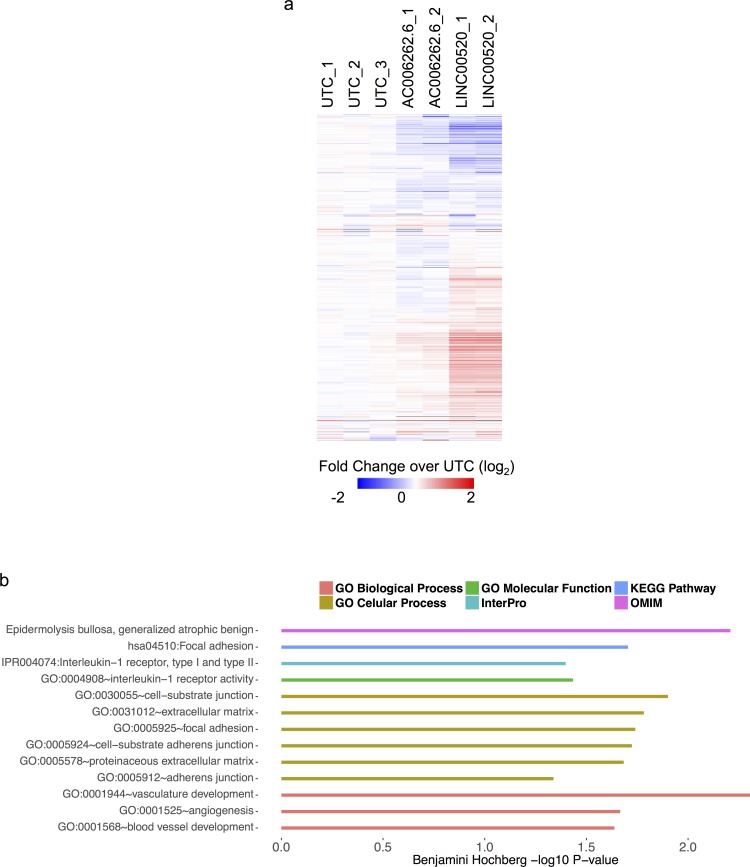
Heat map showing expression profile of MCF10A cells expressing LINC00520 **a.** All genes (1693 genes) depicted in the heat map are upregulated in MCF10A-Src transformed cells. Abbreviations: UTC - Untransformed MCF10A cells; AC006262.6 is a lncRNAs that is not involved in transformation (negative control). Scale bar shows log2 row median centered expression values. **b.** GO analysis of a subset of genes that are upregulated upon ectopic expression of LINC00520 in MCF10A cells (grey line in heat map denotes top 291 genes used for analysis).

Based on the above loss-of-function studies, we next determined whether ectopic expression of LINC00520 in parental WT MCF10A cells leads to gene expression alterations in cancer-associated genes. MCF10A cells were transduced with lentivirus made from a modified lentiviral construct designed specifically for the over-expression of non-coding RNAs [21]. This construct was used for the ectopic for expression of either LINC00520 or a control lncRNA (AC006262.1) that is not expressed in either parental MCF10A-Src, or MCF10A *PIK3CA* (*H1047R*) cells. We compared the expression profiles of untransduced to transduced cells 72 hours post infection using RNA-seq. Using Cuffdiff2 for differential gene expression analysis we first established that LINC00520 was induced upon transduction (~500 fold) and then found that 1,898 genes changed significantly in response to transduction with LINC00520. To determine if LINC00520 might have a regulatory role in transformation we next asked if the upregulated genes during MCF10A-Src transformation were enriched in the genes induced by LINC00520 transduction (976 out of the 1,898). We tested for the enrichment of upregulated genes from each time point using the hypergeometric test (4,12, and 36 hours) and found significant overlap with LINC00520 induction signature for all time points (at 4 hours *p* < 1e-20, at 12 hours *p* < 8e-38 and at 36 hours *p* < 0.008). The most significant overlap was found not at the time point with the highest LINC00520 expression (36 hours) but at 12 hours where LINC00520 expression begins to ramp up in the MCF10A-Src time course (Figure 6A). A minority of the 12-hour MCF10A-Src up-regulated gene set is down regulated in response to LINC00520 overexpression. However most of the changes in either direction with respect to the untransfected control are not observed in the control lncRNA transduction. Interestingly, a subset of these LINC00520-upregulated genes have been linked to cell migration and invasion among other cellular functions (Figure 6B, Supplementary Figure 4). This analysis is consistent with the findings that LINC00520 modulates cell migration in breast cancer cell lines, a phenotype that is recapitulated in *v-Src*-transformed cells.

## DISCUSSION

Here we identify and describe a novel lncRNA, LINC00520, and provide evidence for a potential role in breast cancer. LINC00520 is transcriptionally upregulated in immortalized mammary epithelial cells transformed by oncogenic Src or oncogenic PI3K. We also show that the transcription factor STAT3 is critically required for the regulation of LINC00520 expression. Notably, expression of LINC00520 is increased in basal-like breast cancer cells, which also show a preferential increase in STAT3 activity. Thousands of lncRNAs have been annotated to date yet the biological significance of the majority of these lncRNAs remains unclear. In this study, we provide evidence for a role for LINC00520 in breast cancer. Both loss-of-function and gain-of-function approaches point to a role for LINC00520 in cell migration and cell invasiveness, arguing against the notion that LINC00520 represents a trivial, promiscuous transcript.

To date, no orthologs of LINC00520 have been described in other mammalian species. This is not unexpected, since most lncRNAs appear to be poorly conserved and tend to undergo rapid evolution. For instance, only approximately 12% of human and mouse lncRNAs have orthologs in other species [22, 23] and less than 6% of zebrafish lncRNAs share homologous sequences in humans or mice [24]. While this lack of conservation may suggest lncRNAs are merely cryptic noise in the genome, the characterization of Xist challenges this notion. Although Xist is critical for dosage compensation, most of its sequence is poorly conserved and deletion of exon 4, which shows the most convincing evidence for conservation, has no functional consequence [25]. A more comprehensive catalog of lncRNAs from several vertebrate species along with advancements in computational assessment tools may improve comparative lncRNA studies.

Our study also demonstrates that LINC00520 affects global gene expression. Importantly, ectopic expression of LINC00520 results in a transcriptional profile that shows significant overlap with that generated by Src-transformed cells. LINC00520 might mediate its function by interacting with various chromatin modifiers and/or transcriptional regulators, as observed for lncRNAs such as HOTAIR, HOTTIP, MISTRAL and Xist [24, 26–28]. Despite these examples, it is still unclear whether these RNA-protein interactions are direct. Furthermore, the factors that dictate the specificity of these interactions have not been clearly defined. Regardless, future work will determine the mechanistic action of LINC00520. While there are well-studied examples of lncRNAs that are enriched in the nucleus [29–31], many lncRNAs appear to be cytoplasmic [32–34]. It will be interesting to determine the cellular localization of LINC00520, whether it undergoes any re-localization to various intracellular compartments and how it evades RNA decay.

Despite the definition of lncRNAs as noncoding, nearly half of the expressed lncRNAs encode peptides that are actually translated [35]. Ribosome profiling in ER-Src cells [35] reveals that non-overlapping regions of LINC00520 RNA are translated into 4 peptides (26, 32, 32, and 40 amino acids) that are conserved in monkeys but not in more evolutionary distant species. The stabilities and hence steady-state levels of these peptides are unknown, and whether one or more of these peptides are biologically functional remains to be determined.

In summary, this study supports the accumulating evidence that lncRNAs may function to modulate human cancer pathogenesis. It points to a role for lncRNAs in the mechanism of action of critical oncogenes, namely Src, PI3K and STAT3. To our knowledge, this is the first study that investigates the regulation and biological function of LINC00520. It also implicates the first lncRNA identified as a downstream effector of the PI3K pathway. Future studies will dissect the entire complement of LINC00520 biology and its significance in processes critical for breast cancer initiation and progression.

## MATERIALS AND METHODS

### Antibodies, plasmids and reagents

pBabe puro HA PIK3CA WT (Addgene plasmid #12522) and pBabe puro HA PIK3CA H1047R (Addgene plasmid #12524) was a gift from Dr. Jean Zhao. pBABE-puro was a gift from Hartmut Land, Jay Morgenstern and Robert Weinberg (Addgene plasmid #1764). Isogenic MDA-MB-231 cells expressing PIK3CA WT, PIK3CA E545K and PIK3CA H1047R were a kind gift from Dr. Jonathan Backer, and have been described [36].

### Retroviral expression of LINC00520

LINC00520 was cloned as described previously [21].

### RNA interference

For LINC00520 and PIK3CA shRNAs, single-stranded oligonucleotides sense and antisense pairs, encoding the indicated target sequences were synthesized and cloned into PLKO.1 vector. Stable cell lines were maintained in 2μg/ml puromycin.

PIK3CA shRNA#1, sense, 5′-CCGGGCACAATCCATGAACAGCATTCGAGAATGCTGTTCATGGATTGTGCTTTTTTG-3′;

PIK3CA shRNA#1, antisense, 5′-AATTCAAAAACACAATCCATGAACAGCATTCTCGAGAATGCTGTTCATGGATTGTG-3′

PIK3CA shRNA#2, sense, 5′-CCGGGCATTAGAATTTACAGCAAGACTCGAGTCTTGCTGTAAATTCTAATGCTTTTTTG-3′;

PIK3CA shRNA#2, antisense, 5′-AATTCAAAAAGCATTAGAATTTACAGCAAGACTCGAGTCTTGCTGTAAATTCTAATGC-3′

LINC00520 shRNA#1, sense, 5′- ccggAAGAGAAAAGCTGAAGGACACctcgagGTGTCCTTCAGCTTTTCTCTTtttttg

LINC00520 shRNA#1, antisense, 5′- ccggAAGAGAAAAGCTGAGAAGGACACctcgagGTGTCCTTCAGCTTTTCTCTT

LINC00520 shRNA#2, sense, 5′ - ccggACCTCAAATCTTTCGAGAACActcgagTGTTCTCGAAAGATTTGAGGTtttttg

LINC00520 shRNA#2, antisense, 5′- AATTCAAAAAACCTCAAATCTTTCGAGAACActcg agTGTTCTCGAAAGATTTGAGGT

### Cell culture and immunoblotting

MCF10A and MCF10DCIS.com [37] cells were cultured in Ham's F12/DMEM (Cellgro) supplemented with 5% equine serum (Cellgro), 500ng/ml hydrocortisone (Sigma-Aldrich), 100ng/ml cholera toxin (List Biological Laboratories), 20ng/ml EGF (R&D Systems) and 10ug/ml insulin. SKBR3 cells were maintained in 10% FBS/McCoy's (Cellgro). MCF7, and MDA-MB-468, MDA-MB-453, MDA-MB-231 cells were maintained in Dulbecco's modified Eagle medium (DMEM; Cellgro) supplemented with 10% Fetal Bovine Serum (FBS; Cyclone). T47D and BT549 cells were cultured in 10% FBS/DMEM, supplemented with 1mg/ml insulin (Sigma-Aldrich). SUM159-PT cells were grown in Ham's F12 medium (Cellgro) supplemented with 5% FBS, 1ug/ml hydrocortisone (Sigma-Aldrich) and 5ug/ml insulin (Sigma-Aldrich). ZR75-1 and HCC1806 were maintained in RPMI 1640 supplemented with 10% FBS (Cellgro). MCF10A-Src inducible cell lines were cultured as previously described [17]. For all western blotting, cells were lysed in RIPA buffer with protease and phosphatase inhibitors.

### 3D Morphogenesis assay

MCF10DCIS cells were grown in three dimensional Matrigel cultures as described [38]. Briefly, 3x10^3^ cells were suspended in modified growth medium containing 2% growth factor-reduced Matrigel, 2% Horse serum (Cellgro) and 5ng/ml EGF (R&D systems). Cell mixture was plated on top of a solidified layer of growth factor-reduced Matrigel. Cells were fed every 4 days. Phase contrast images were acquired using the Nikon Eclipse Ti microscope.

### Quantitative real-time RT PCR

Total RNA was isolated using the RNeasy kit following the manufacturer's instructions (Qiagen). Reverse transcription was performed using Quantitect Reverse transcription kit according to the manufacturer's instructions (Qiagen). Quantitative real-time RT-PCR was performed using SYBR Green PCR Master Mix (BioRad) and the ABI Prism 7900 sequence detector (Applied Biosystems). Relative mRNA expression was calculated by the ΔΔCT method with GAPDH as reference. Primer sequences:

LINC00520 sense: 5′- GTGTACATTTCTGGGTAGCTT

LINC00520 antisense: 5′ - AAAGGAAAACAATACAGGCTTG

GAPDH sense: 5′ - GCAAATTCCATGGCACCGT

GAPDH antisense: 5′- TCGCCCCACTTGATTTTGG

### Transwell migration assay

Transwell migration assay using SUM159-PT cells was performed as previously described [39]. Cells were allowed to migrate through an 8μm-pore transwell (Corning) for 16 hours and NIH3T3 conditioned media was used as a chemoattractant in the lower chamber. MCF10A-Src-Hygro inducible cells containing LINC00520 shRNA constructs were first treated with 1μM Tamoxifen or ethanol (vehicle) for 48 hours prior to migration. Cells were then serum starved and then allowed to migrate for 16 hours using MCF10A growth media as the chemoattractant. MCF10A-Src-Hygro inducible cell lines were generated using a retroviral pLHCX vector (Clontech) containing an ER-Src inducible gene. Stable cell lines were established after selection with 150μg/ml hygromycin for 5 days and maintained thereafter in 50μg/ml.

### RNA-Seq analysis and ChIP-Seq analysis

RNA whole transcriptome sequencing (RNA-seq) was carried out at 0 (1 replicate), 4 (2 replicates), 12 (1 replicate) and 36hrs (2 replicates) post tamoxifen treatment on polyA selected RNA using Illumina tru-seq library construction. RNA-seq was also carried out for the over-expression experiments in parental MCF10A cells that were untransduced (3 replicates), transduced with LINC00520 (2 replicates) or transduced with a control lincRNA AC006262.6 (2 replicates) on polyA selected RNA using Illumina tru-seq library construction harvested 72 hours post transduction.

Transcript levels were quantified and differentially expressed genes were called using cuffdiff2 [40]. Relative transcript levels are expressed as a “Fragments Per Kilobase of transcript per Million mapped” (FPKM) which corrects for the length of the transcript and the depth of the libraries. Raw and processed ChIP-Seq data was retrieved from [18].

### Expression analysis in clinical samples of human breast invasive carcinoma

Genomic alterations associated with LINC00520 were identified by querying genomic data from Breast Invasive Carcinomas dataset of TCGA. Data from 850 breast cancer cases were retrieved from the TCGA database (http://cancergenome.nih.gov/), including RNAseq gene expression (Illumina HiSeq RNASeqV2 Level 3.1.9.0) and lncRNA expression from TANRIC database (http://bioinformatics.mdanderson.org/main/TANRIC:Overview). Intrinsic molecular subtype data was obtained by applying the PAM50 algorithm to the RNA-seq data.

LINC00520 expression level was compared in normal-tumor paired samples (*n* = 105) using a *T*-test for paired samples and compared using One-way ANOVA (with unequal variance) in carcinoma samples (*n* = 741) by intrinsic molecular subtypes. Afterwards, LINC00520 expression levels were dichotomized into low and high expression categories using univariate clustering based on finite normal mixture modeling (mclust 5.1 package, R 3.2.2). Differential gene expression was performed using two-class unpaired significance of microarray analysis (SAM 2.0 package, R 3.2.2) [41] in basal carcinomas previously classified with low (*n* = 96) and high (*n* = 30) expression of LINC00520. Next, we performed a pre-ranked GSEA (Gene-Set Enrichment Analysis) using software provided by the Broad Institute (http://www.broadinstitute.org/gsea/msigdb/annotate.jsp) on a gene list ranked based on the d-statistic computed from the LINC00520 differential expression analysis, and we assessed enrichment using the Broad Institute's Molecular Signatures Database (mSigDB) Hallmark gene sets collection (*n* = 50) (http://www.broadinstitute.org/gsea/msigdb/). Finally, functional annotation for the transcriptomic signature of basal-like high LINC00520 expression carcinomas was performed using Database for Annotation, Visualization and Integrated Discovery (DAVID) 6.7 [42]. Gene set enrichments were assessed using Gene Ontology Biological Pathway and KEGG Pathways summarized version with 13,015 genes (total genes in RNASeqV2 data) as background list and gene sets containing a minimum of 15 genes. Statistical significance was considered when *p*-value was < 0.05 or false discovery rate (FDR) was < 0.05.

### Sulforhodamine B (SRB) proliferation assay

For proliferation assay 0.01 x 10^6 cells were seeded in complete growth media. Relative number of adherent cells at day 0 (~16hrs post-seeding), days 1, 3, and 4, were assessed using sulforhodamine B assay as previously described [43]. Briefly, adherent cells were fixed with 12.5% (w/v) trichloroacetic acid for 1 hour at 4°C. Cells were then rinsed three times with water and stained with a solution of 0.5% (w/v) SRB in 1% acetic acid for at least 30 minutes at room temperature. Cells were then washed three times with 1% acetic acid and allowed to dry. SRB was dissolved in 10 mmol/l Tris (pH 10.5). Absorbance of solubilized SRB was measured at 510 nm.

## SUPPLEMENTARY MATERIAL








