# Supplementary material for: LINC00520 is induced by Src, STAT3, and PI3K and plays a functional role in breast cancer
Source: Oncotarget. 2016 Sep 10;7(50):81981–94. doi: 10.18632/oncotarget.11962 (PMC5347668; doi:10.18632/oncotarget.11962)
Supplement: Supplementary file 1 [file oncotarget-07-81981-s001.pdf]

LINC00520 is induced by Src, STAT3, and PI3K and plays a functional role in breast cancer

Supplementary Material

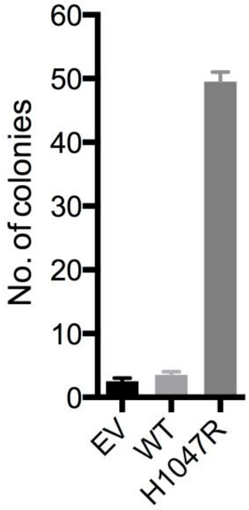

Supplementary Figure 1. Colony growth in soft agar, MCF10A cells stably expressing *PIK3CA* wild-type and mutant *PIK3CA H1047R* were compared relative to empty vector control (EV). Statistical significance was determined using paired Student's t-test, * p<0.05, ** p<0.01, ***p<0.001. For colony formation in soft agar, $5x10^4$ cells were suspended in MCF10A growth media and 0.4% noble agar. Cell suspension was plated on top a solidified layer of 0.8% noble agar. Cells were fed with growth media every 4 days. After 21 days, colonies were stained with 1mg/ml iodonitrotetrazolum chloride and quantified.
